# Incipient Diabetes Treated with Long-Term Classical Prescription

**DOI:** 10.1155/2019/3054213

**Published:** 2019-11-05

**Authors:** Xiuxiu Wei, Jiaxing Tian, Xinmiao Wang, Haoran Wu, Haiyu Zhang, Xiaolin Tong

**Affiliations:** ^1^Department of Endocrinology, Guang'anmen Hospital, China Academy of Chinese Medical Sciences, Beijing 100053, China; ^2^Graduate College, Beijing University of Traditional Chinese Medicine, Beijing 100029, China

## Abstract

**Background:**

Diabetes mellitus (DM) belongs to the progressive and irreversible disease. With the development of the disease, the function of beta cells declines significantly. Current treatments cannot reverse the course of the disease. The role of traditional Chinese medicine (TCM) in the DM treatment has been recognized widely, while there are few long-term observation reports. In this study, we introduced a case of DM treated by classical prescription alone for 10 years, which would provide the reference for clinical practice.

**Case Presentation:**

A 64-year-old male complained of a 2-month history of dry mouth, frequent urination, and blurred vision and found increased blood glucose for 3 days. The patient's glycated hemoglobin was 14.2%, fasting plasma glucose fluctuated at 12-15 mmol/L, and urinary albumin excretion rate (UAER) was 32.9 *μ*g/min. The male was treated with 10 years of continuous classical prescription alone. After 3 months of TCM treatment, the patient's blood glucose level decreased significantly and blurred vision symptoms improved. With continued TCM treatment, his UAER normalized. Subsequently, he continued outpatient consultation, and his TCM prescription was adjusted according to clinical symptoms. After 10 years of continuous TCM treatment, his blood glucose remained stable, urinary microalbumin quantitation showed no abnormalities, and blurred vision disappeared.

**Conclusions:**

This case provides specific treatment plans and effective references for long-term control of blood glucose, prevention and treatment of diabetes complications, delay of disease progression, and protection of impaired islet function in the treatment of diabetes with TCM. TCM may become a meaningful alternative DM treatment in the future.

## 1. Background

According to the International Diabetes Federation in 2017, nearly 9.9% of the world's population or 693 million people will be suffering from diabetes mellitus (DM) by 2045 [1]. With the aging of the population and changes in lifestyle in China, diabetes has reached a prevalence of 10.9%, accounting for one-third of the total number of diabetic patients in the world [2]. DM can cause severe microvascular and macrovascular complications. Within 10 years after the diagnosis of diabetes, diabetic neuropathy is found in 60%-90% of cases [3], and 40% of end-stage renal disease cases are due to diabetes in the United States [4]. Furthermore, more than 60% of patients with diabetes develop retinopathy within 10 years, approaching nearly 100% by 15 years [5]. Given the chronic and severe complications of diabetes, the disease carries a heavy social, financial, and healthcare systems burden. With a prolonged course, pancreatic beta cells are gradually destroyed, insulin synthesis and secretion are permanently reduced, and islet dysfunction gradually becomes irreversible [6].

However, the clinical treatment and control of T2DM and its complications have unsatisfactory side effects. Glyburide causes hypoglycemia significantly more frequently than other oral medications [7]. Sulfonylureas, particularly glimepiride, have the highest proportion of adverse reactions (up to 32.5%), mainly severe hypoglycemia. The next highest proportion of adverse reactions are reported for acarbose (17.2%), mainly gastrointestinal reactions. Pioglitazone (11.3%) mainly causes damage to the cardiovascular system. Other adverse drug reactions include lactic acidosis caused by biguanides, edema, and weight gain caused by thiazolidinediones, such as rosiglitazone [8]. It has been reported that although patients taking empagliflozin/linagliptin show significant hypoglycemic effects without developing severe hypoglycemia, they carry a high risk of urinary tract and genital infections [9]. Therefore, it is important to explore safer and effective treatment methods.

There is a long history in treating DM by traditional Chinese medicine (TCM). Serious of large-scale clinical randomized controlled trials (RCTs) have demonstrated the efficacy of TCM in antidiabetes and improving islet function. Dachaihu decoction and Gegen Qinlian decoction, as classical prescriptions, have shown that they are effective in controlling blood glucose level and are safe to use in DM patients by RCTs [10, 11]. A retrospective study of 142 diabetic patients for at least 6 months proved that classical prescription could reduce the use of antidiabetes drugs. In addition, Chinese herbal decoction significantly enhanced the hypoglycemic action and had a certain effect on protecting islet cell function [12]. A 12-week randomized double-blind controlled trial of 186 patients with diabetes mellitus showed that the Chinese herbal medicine Jinlida significantly improved hypoglycemic action compared with placebo on the basis of metformin. Furthermore, Jinlida showed improved *β*-cell function with a HOMA-*β* increase [13, 14]. The study included 800 patients with unsatisfactory glycemic control; treatment with Xiaoke Pill led to significant reduction in the risk of hypoglycemia and similar improvements in glycemic control compared to glibenclamide [15]. However, we did not have a long enough observation course in clinical trials.

This paper describes a case of primary T2DM treated with classical prescription alone for 10 years; the treatment significantly reduced blood glucose, normalized urinary protein levels, improved blurred vision, controlled the occurrence of vascular complications, protected damaged islet function, and delayed the course of diabetes. Blood glucose levels were well controlled after a long-term follow-up treatment. Once the patient's condition was stabilized, the form of TCM powder, which is suitable for long-term use, was adopted as it was easy to prepare, which enhanced compliance and achieved good clinical efficacy.

## 2. Case Presentation

A male patient (age: 64 years, height: 171 cm, weight: 72 kg, body mass index: 24.6 kg/m^2^) first visited our endocrinology clinic in November 2007 after having been diagnosed with T2DM 3 days earlier at another hospital. At that visit, his glycated hemoglobin (HbA1c) was 14.2%, fasting plasma glucose (FPG) was 14.7 mmol/L, and urinary albumin excretion rate (UAER) was 32.9 *μ*g/min. Routine urine analysis showed GLU (+) 5.5 mmol/L. A fundus examination yielded negative results. Before diagnosis, he had polydipsia, polyuria, and weight loss. When he visited our clinic, he complained of dry mouth and blurred vision and took oral medication for hypoglycemia for only 1 day (acarbose 50 mg and glimepiride 1 mg, 3 times a day).

We advised the patient to stop taking oral hypoglycemic drugs and change to TCM treatment. We prescribed a formula consisting of Chaihu (*Radix Bupleuri*) 15 g, Huangqin (*Radix Scutellariae Baicalensis*) 30 g, Qingbanxia (*Pinelliae Rhizoma Praeparatum Cum Alumine*) 15 g, Zhishi (*Fructus Aurantii Immaturus*) 15 g, Dahuang (*Radix Et Rhizoma Rhei Palmati*) 3 g, Huanglian (*Rhizoma Coptidis*) 45 g, Ganjiang *(Rhizoma Zingiberis Recens*) 9 g, and Zhimu (*Common Anemarrhena Rhizome*) 30 g.

The patient returned for follow-up after 1 month of treatment. His HbA1c was 11.0% and UAER was 31.53 *μ*g/min. During the first 2 weeks of the medication, his FPG fluctuated between 6.7 and 9.2 mmol/L and his 2 h plasma glucose (2hPG) fluctuated between 5.1 and 13.3 mmol/L. In the subsequent 2 weeks, his FPG fluctuated between 6.0 and 7.4 mmol/L and 2hPG fluctuated between 4.9 and 9.6 mmol/L. His blurred vision was relieved. As the patient still reported symptoms of dry mouth and early awakening, we added Suanzaoren (*Ziziphi Spinosae Semen*) 30 g, Shuizhi (*Hirudo*) 9 g, and Chishao (*Radix Paeoniae Rubra*) 30 g to the original preparation.

In January 2008, the patient returned for follow-up after another month of the modified treatment. His HbA1c was 7.1%, FPG was 6.4 mmol/L, 2hPG was 7.1 mmol/L, and UAER was 17.11 *μ*g/min (within the normal range). Moreover, his sleep symptoms improved.

In February 2008, the patient visited our hospital for the fourth time. His FPG was 5.3 mmol/L, 2hPG was 7.7 mmol/L, and bedtime blood glucose was 8.2 mmol/L. The patient's blurred vision had disappeared, and he had no other particular discomfort. We adjusted the prescription to Zhimu (*Common Anemarrhena Rhizome*) 30 g, Huangqin (*Radix Scutellariae Baicalensis*) 15 g, Huanglian (*Rhizoma Coptidis*) 30 g, Rougui (*Cinnamomum cassia Presl*) 15 g, Shengdihuang (*Rehmanniae Radix*) 60 g, Shuizhi (*Hirudo*) 9 g, Suanzaoren (*Ziziphi Spinosae Semen*) 30 g, and Wuweizi (*Fructus Schisandra Chinensis*) 9 g. The prescription was powdered and prescribed to be taken twice a day, 9 g each time.

From March 2008 to February 2011, the patient was seen at follow-up visits 9 times. Additional herbal medicine was added or subtracted on the basis of the fourth prescription. The patient's blood glucose was stably controlled, and HbA1c fluctuated between 4.9% and 6.2%. On March 7, 2011, the patient visited the hospital for the 16^th^ time; his HbA1c was 5.6%, FPG was 6.1 mmol/L, and 2hPG was 7.9 mmol/L. The patient continued to use the powdered prescription orally.

From April 2011 to January 2015, the patient was followed up another 5 times, during which further modifications were made to the prescription at the 16^th^ visit. The patient's blood glucose control remained stable, and the HbA1c fluctuated between 5.6% and 6.5%. His urinary microalbumin/urinary creatinine was 8.31 mg/g. Fundus examination yielded a negative result for retinopathy.

From June 2016 to June 2018, the patient was followed up 3 times. His main symptom was nocturia (once or twice per night). Considering that the patient is an elderly male and given the chronic course of diabetes, we prescribed a formula consisting of Tianma (*Rhizoma Gastrodiae*) 180 g, Roucongrong (*Cistanche deserticola*) 540 g, Ganjiang *(Rhizoma Zingiberis Recens*) 180 g, Huanglian (*Rhizoma Coptidis*) 540 g, Zhimu (*Common Anemarrhena Rhizome*) 540 g, Chishao (*Radix Paeoniae Rubra*) 540 g, Shanzhuyu (*Fructus Macrocarpii*) 540 g, Tianhuafen (*Radix Trichosanthis*) 540 g, Xiyangshen (*Panax quinquefolium*) 540 g, and Sanqi (*Panax notoginseng*) 270 g. The prescription was powdered and prescribed to be taken twice a day (9 g each time). The blood glucose remained stable over a long period, UAER showed no abnormalities, and blurred vision had not returned.

The patient was reviewed every 3 months by telephone follow-up. His HbA1c fluctuated in the range of 6.1%-6.5%, FPG fluctuated in the range of 6.5-7 mmol/L, 2hPG remained <10 mmol/L, and urinary microalbumin/urinary creatinine remained normal over the long term.

**Figure 1:**
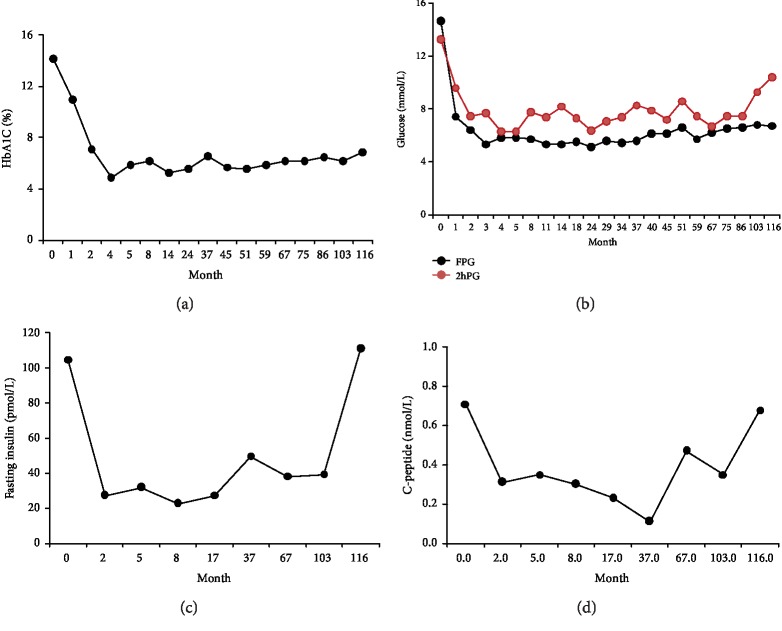
Intervention of TCM decreased blood glucose and improved islet function. (a) Changes in HbA1c: the level of HbA1c decreased significantly after the initial 3 months of treatment and showed a downward trend that tended to be stable after the long-term treatment. (b) Changes in glucose: the level of FPG and 2hPG showed a downward trend and fluctuated within the control standard range. (c) Changes in fasting insulin: the level of fasting insulin improved after the long-term treatment. (d) Changes in C-peptide: the level of C-peptide improved after the long-term treatment. TCM: traditional Chinese medicine; HbA1c: glycosylated hemoglobin; FPG: fasting plasma glucose; 2hPG: 2-hour postprandial blood glucose.

The patient is now followed up regularly in our clinic. During follow-up, blood glucose changes (Figure 1; Supplementary [Supplementary-material supplementary-material-1]) and islet function (Supplementary [Supplementary-material supplementary-material-1]) are reviewed. Blood glucose and vascular complications remained stably controlled. During the 10 years since the initial visit, the patient had not used any oral medication or insulin for blood glucose control, and his HbA1c had reached normal levels by using the TCM powder alone long term. The classical prescription is based on the natural evolution of diabetes, “prediabetes, diabetes, advanced diabetes, and diabetic complications.” According to the changes in the patients' clinical symptoms and the characteristics of the chronic disease course, drugs should be adjusted to select the appropriate TCM with hypoglycemic effects. The HbA1c and blood glucose levels normalized within a short period. We added the herbal medicine to improve circulation during the early stage of treatment to reverse diabetic vascular complications, return urinary microalbumin levels to normal, and improve blurred vision. The patient took TCM to improve hypoglycemia and circulation for a long time, without any adverse reactions or new complications. Medical history and reports in this study had been approved by the patient.

## 3. Discussion and Conclusion

The patient presented here had high blood glucose levels when he was diagnosed with DM (HbA1c, 14.2%); he also had microalbuminuria and blurred vision caused by elevated blood glucose levels. The patient visited our clinic after receiving oral hypoglycemic drugs for 1 day only, after which he was asked to switch to TCM alone. The patient's blood glucose levels were significantly reduced, microalbuminuria turned to normal, and blurred vision was improved with classical prescription. Although there were signs of clinical proteinuria with renal function injury at the initial diagnosis, the urine protein normalized rapidly after taking the oral decoction and remained normal during follow-up. The TCM was then prepared as a powder, and the patient took this with good compliance. During the follow-up treatment with TCM alone for 10 years, his blood glucose control was stable, the urine microalbumin test showed no abnormalities, and no fundus lesions appeared. The patient did not use western medicine, and no adverse reactions such as hypoglycemia occurred. Such cases have rarely been reported. Therefore, this report may provide a reference for the treatment of T2DM in lowering blood glucose and preventing and treating complications with TCM alone.

T2DM is a progressive metabolic disorder characterized by elevated blood glucose and is characterized by insulin resistance to glucose metabolism, with multiorgan involvement. According to previous studies, the pathophysiological process of diabetes included lipid metabolism disorder, decreased effect of insulin-stimulating hormone, increased basal glucagon level, renal dysregulation, and neurotransmitter dysfunction [16]. Based on the physiological and pathological process of T2DM, western medical treatments range from hypoglycemic drugs, such as sulfonylureas, biguanides, and insulin, to newly developed dipeptidyl peptidase IV inhibitors, glucagon-like peptide-1 receptor agonist, sodium-glucose cotransporter-2 inhibitor, and various insulin analogs. Bariatric surgery and other interventional treatments affect afferent vagus nerve activity- (entero-brain axis-) related control of internal organs, such as the liver and pancreas, to provide obesity-related metabolic control of T2DM [17]. However, long-term oral hypoglycemic drugs or insulin are resistant, and newly developed drugs aggravate the economic burden of patients and easily lead to poor patient compliance. The mechanism of obesity-related T2DM surgery remains unclear, has major limitations, and has not been widely performed. The use of many oral hypoglycemic drugs is limited due to the occurrence of diabetic nephropathy, and insulin therapy also increases body weight, which indirectly increases the risk of diabetic complications. Although these treatments can control blood glucose within the target range, it cannot reverse the process of islet cell failure, and the effects on the associated complications are not obvious.

Studies have suggested that TCM treatment for T2DM is effective, particularly in terms of prevention and reversal of multimetabolic disorders, such as obesity, blood lipids, blood pressure, and fatty liver caused by diabetes [10, 18, 19]. This case reported here had a significantly elevated HbA1c level and diabetic nephropathy urinary protein level, which returned to normal within a short time after the initial 3 months of treatment with TCM only. Once HbA1c was controlled within the target range, the prescription was changed to TCM powder. HbA1c continued a downward trend and stabilized at normal levels with long-term treatment. Furthermore, TCM improved the symptoms of blurred vision, control the occurrence of vascular complications, improved the impaired islet function, and delayed the course of DM.

The ancient Chinese medicine book *Huangdi Neijing* has recorded the diabetes, while diabetes is thought to be caused by obesity nowadays to a great extent and is divided into four stages: prestage, diabetes stage, middle-late stage, and complication stage. The appropriate TCM should be chosen based on the characteristics of different stages. In a large-scale clinical trial, we have previously found that the classical prescription, Gegen Qinlian decoction, has an independent hypoglycemic effect. Compared with placebo, this decoction reduced HbA1c significantly, and the hypoglycemic effect was positively correlated with the dose used. Gegen Qilian decoction has been shown to reduce glucose by regulating gut microbiota and improving islet function, and its hypoglycemic effect remained after drug withdrawal [11]. In addition, TCM can reduce the risk of complications by addressing multiple targets while reducing blood glucose. In a randomized controlled trial including T2DM patients with abdominal obesity and dyslipidemia, TCM had equivalent efficacy to metformin in lowering glucose and was superior to metformin in improving lipid levels and waist circumference [19].

Due to the multiple vascular complications of diabetes, it has been proposed that DM should be called “TangLuo disease,” given the damage to collaterals caused by elevated blood glucose [20]. TCM treatment of DM emphasizes the early improvement of the circulation and is maintained throughout the treatment. This can effectively prevent the occurrence of diabetic complications. In the early stage of treatment, we used drugs to improve circulation; these included Sanqi (*Panax notoginseng*) and Shuizhi (*Hirudo powder*). The extracts of Sanqi (*Panax notoginseng*) have strong anti-inflammatory and antioxidant effects and protect the vascular endothelium [21, 22]. Shuizhi (*Hirudo powder*) is an animal-derived medicine, which has anticoagulant effects [23]. Shuizhi (*Hirudo powder*) combined with Huangqi (*Radix Astragali Mongolici*) is used to treat diabetic nephropathy, as these herbal medicine can protect kidney function, enhance insulin sensitivity, have antioxidant activity, and improve mitochondrial function [24].

With the increasing incidence of obesity, T2DM may become more prevalent in the future. DM has a significant impact on the quality of life of patients and imposes a heavy burden on the finance and public health care system. The use of TCM for treating DM is increasingly gaining attention, with clinical trials demonstrating that herbal medicine can lower blood glucose and provide additional benefits, such as improving insulin resistance and reducing lipid levels and weight [13–15, 25], and pharmacological studies showing that TCM can improve islet function, increase insulin secretion, and enhance the use of glucose in peripheral tissues [26–31]. TCM can also be used to treat diabetic complications through improving blood viscosity, microcirculation, and oxidative stress-related abnormalities [32].

Once our patient's condition was stable and blood glucose levels had normalized, we adjusted the prescription to a powder to allow oral administration. Such form is convenient for patients to carry with them, improving long-term medication compliance. In addition, it can effectively prevent and delay the occurrence and progression of complications [33]. The configuration of TCM (powder, formula granule, or pill) can be adjusted flexibly for long-term use, which is more conducive to patient compliance and long-term treatment.

DM is an important chronic, noncommunicable disease. Its treatment and complications not only place a marked economic burden on patients but also affect their quality of life. The high incidence of diabetes requires development of a new therapeutic strategy. Early detection and early treatment should be emphasized, and circulatory drugs should be used to improve the prevention and treatment of early complications.

There have been few reports on the treatment of DM with TCM alone. This case report provides evidence of the effective regulation of DM, and reversal and prevention of complications, by using classical prescription alone, and provides a reference for clinical application of TCM treatment. Further studies are needed to confirm whether long-term treatment of TCM can reduce the risk of diabetes complications as well as the safety of long-term TCM application. Additionally, the hypoglycemic, circulatory, and protection for islet function of TCM should be clarified. Nevertheless, this study indicates that TCM may be an important alternative therapy for the prevention and treatment of DM and its complications.

## Data Availability

The data that support the findings of this study are stored in Guang'anmen Hospital (Beijing, China) and available from the corresponding authors on reasonable request.

## Abbreviations

DM: Diabetes mellitus
FPG: Fasting plasma glucose
HbA1c: Glycated hemoglobin
2hPG: 2 h plasma glucose
TCM: Traditional Chinese medicine
T2DM: Type 2 diabetes mellitus
UAER: Urinary albumin excretion rate.

## Supplementary Materials

Supplementary Materials Changes in islet function and blood glucose during treatment.
